# Annotations

**Published:** 1903-07-25

**Authors:** 


### ANNOTATIONS.

### The Northampton General Hospital.

It is sound testimony to the growth of hospital opinion throughout the country that probably the largest and most influential meeting of governors ever held in connection with this institution should have abolished the open board system with practical unanimity. The management will henceforth be vested in a committee, of which the honorary physicians and surgeons will be ex-officio members. This radical change in the constitution must be followed in the near future by a re-arrangement of the duties of various resident officers and a re-organisation of the staff. The new hospital buildings, which promise to be the cheapest and probably the best of their kind, will be ready for occupation by the beginning of December. The new building occupies an excellent site and is well adapted for the treatment of cases of tuberculosis. The floors are teak throughout, a wise provision, as this material is the best for the purpose. The builders have done their work well, and providing the equipment and furnishing are carefully and wisely carried out this hospital should prove a model of efficiency. We congratulate the governors upon the good work which they have in hand and the thoroughness which has marked their proceedings throughout. The Northampton General Hospital, reconstituted and rebuilt, has before it a career full of promise for the future.

### The Open Spaces of North London.

The residents of Hampstead have shown praiseworthy forethought in the matter of open spaces. With their ancient Heath as a nucleus they have kept a considerable area of rolling meadow from the builder on the Highgate side, and have secured the charming demesne of Golder's Hill for the public use on the northern limit. They are now anxious to add some 80 acres more on the north-west corner of the Heath. The immediate occasion is the making of a new tube railway from Charing Cross to Hampstead by Euston, which is expected to call into being a working-class suburb around its terminus on the Finchley Road, as well as to bring an influx of holiday-makers to the Heath itself. The Eton College Trustees, who are the landlords, are willing to sell the fields on the far side of the new tube station for the extension of the Heath at a sum which is considered to be generously moderate, namely, £48,000. This works out at about £600 an acre, or about half the price which was paid 14 years ago for Parliament Fields, and £400 an acre less than was paid for the Golder's Hill Estate. Active measures are already on foot to raise the money required by public subscription, and a large council has been formed with this end in view, to which many of the most public-spirited and far-seeing members of the community have lent their names. The whole question of open spaces in London is at the present day one of the greatest moment owing to the rapid expansion of the Metropolis. A century hence every acre of free space will be appreciated, and Hampstead Heath, with all the addition that it is now proposed to make to it, will not be any too large as a place of recreation or as a breezy "lung." The public health of London has now happily come to the same level as that of the country-town, and the gratifying result is due in a large measure to the dispersion of the population and to the open spaces. One of the best tests of urban healthiness is the rate of mortality among infants in the hot weather of summer and autumn. In eighteenth-century London the mortality under that head was enormous, and almost peculiar to the capital, but at present it is only a little higher than the mean of all England and Wales. It is the duty of every citizen to see to it that this high standard of health is maintained and advanced more nearly to the ideal, and he cannot for this purpose do better than lend his hearty support toward maintaining and extending the open spaces of London.

### Rowton Family Homes.

The failure of municipal effort, in London and elsewhere, to provide houses for the poorest is now so generally admitted that those interested in the question are looking around for some new method of solving the difficulty. One suggestion is the building of homes which may be let without loss at a rent of not more than 4s. a week, and which will yet meet all necessary requirements of comfort and decency. The writer thinks that the thing could be done if some of the communal features of the Rowton Houses were adapted to family use. One of his suggestions —the supply of hot water from a common source— is, we think, already carried out in the Guinness buildings, and another, the common kitchen for the cooking of at least the chief meal of the day, is a regular feature of Sunday life in our poorer districts. The weekly joint and potatoes is almost invariably cooked by a neighbouring baker. To make this a daily instead of a weekly occurrence would not be too great a change even for a class that is assuredly exceedingly conservative. With a caretaker to supervise the condition in which they are kept, it would be possible, the writer thinks, to make the number of sanitary conveniences fewer without injury to health. The caretaker's task would, we fear, be no sinecure, and a generous water-supply would be essential if sufficient cleanliness were to be possible. The demand that the living room should be "a place where a family could live without feeling that it is a kitchen, scullery, parlour and bedroom in one" is, however, rather difficult to comply with. Dishes must be washed somewhere, and he would indeed be a utopian who would expect the dames of a whole tenement to wash up in a common scullery without great differences of opinion arising. And it is doubtful if the minor meals of the day will be cooked outside the home. But the suggestion that the sleeping accommodation should be divided up into cubicles, with partitions sufficiently high for decency and privacy, but not so high as to prevent through ventilation, is a sensible one. This would certainly be more healthy than the present choice between small ill-ventilated cupboards to sleep in and the promiscuity of a larger apartment. If all this could be provided without loss to the community for four shillings a week, it would certainly be a boon to the really poor.

